# Papillary Adenocarcinoma of Rete Testis Mimics Inflammatory Lump: A Case Report

**DOI:** 10.1155/2011/857812

**Published:** 2011-09-29

**Authors:** Che-An Wu, Yung-Hsiang Chen, Kee-Ming Man, Jui-Lung Shen, Wen-Chi Chen

**Affiliations:** ^1^Department of Urology, Lee's General Hospital, Da-Chia, 43748 Taichung, Taiwan; ^2^Graduate Institute of Integrated Medicine, College of Chinese Medicine, China Medical University, Taichung 40402, Taiwan; ^3^Department of Urology and Department of Medical Research, China Medical University Hospital, Taichung 40402, Taiwan; ^4^Department of Anesthesiology, Tungs' Taichung Metroharbor Hospital, Taichung 43344, Taiwan; ^5^Department of Dermatology, Taichung Veterans General Hospital, Taichung 40705, Taiwan

## Abstract

We presented a rare extratesticular neoplasm, papillary adenocarcinoma of rete testis, which manifested variable symptoms and mimicked most frequently seen benign extratesticular lesions. Due to its rarity, the treatment is therefore uncertain. Our patient's clinical manifestations mimicked an inflammatory lump and underwent radical orchiectomy after pathological report had been confirmed. Unlike other reports, our patient survives and has a good outcome. No definite predictor and tumor marker can be used to define the prognosis. Early diagnosis and surgical treatment may have a good outcome.

## 1. Introduction

Papillary adenocarcinoma of rete testis is an extremely rare extratesticular neoplasm with only a few sporadic reports in the literature [1]. The clinical manifestations of this tumor may be variables and similar to most frequently seen benign extratesticular lesions that make the diagnosis may be delayed [2]. Due to its rarity, the treatment is therefore uncertain. We reported a young patient with papillary adenocarcinoma of rete testis whose clinical manifestations mimicked an inflammatory lump. We also reviewed the literatures and reported its outcome.

## 2. Case Report

**Figure 1:**
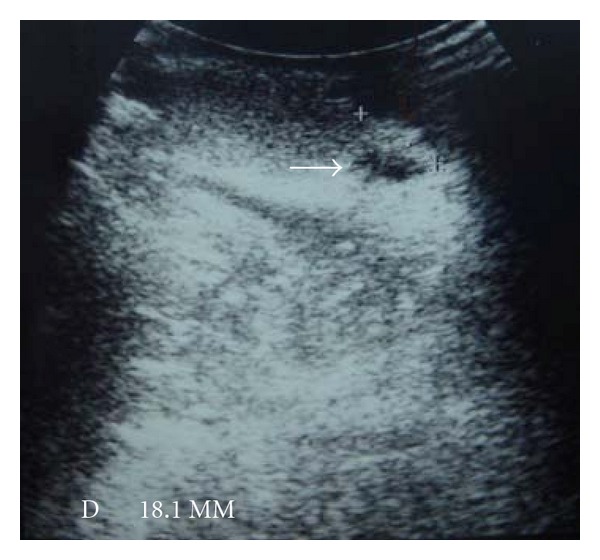
Scrotal ultrasound showing a focal cystic paratesticular mass with compressed testicular parenchyma (arrowhead).

A 30-year-old male patient came to outpatient department presented with a swelling and tender left scrotal lump for 2 weeks. There were associated symptoms of dysuria and frequency in urination within these days. He did not have fever nor body weight loss. Physical examination revealed a hard nodule at the head of left epididymis with severe tenderness. Ultrasonography of the scrotum revealed a 1.6 cm hyperechoic lesion over left epididymis (Figure 1). The initial diagnosis was left epididymitis with an extratesticular lesion. He underwent a partial epididymectomy with removal of the extratesticular lesion in a transverse left scrotal incision under spinal anesthesia after failed medical treatment for 1 week with vibramycin and acetaminophen. During operation, this hard mass was measured about 8 mm located on the head of epididymis with another papillary solid nodule about 7 mm protruded just between the left epididymis and testis. There was a swollen left epididymis found during operation.

**Figure 2:**
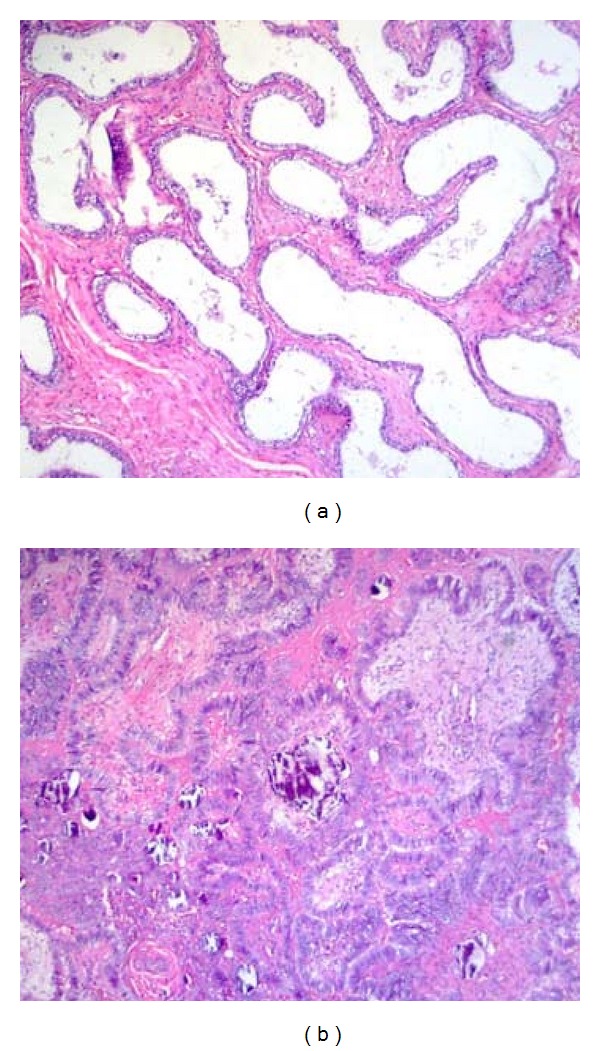
Histological H&E stain of (a) normal microscopic pictures of rete testis, and (b) hyperchromatic cells (100x).

**Figure 3:**
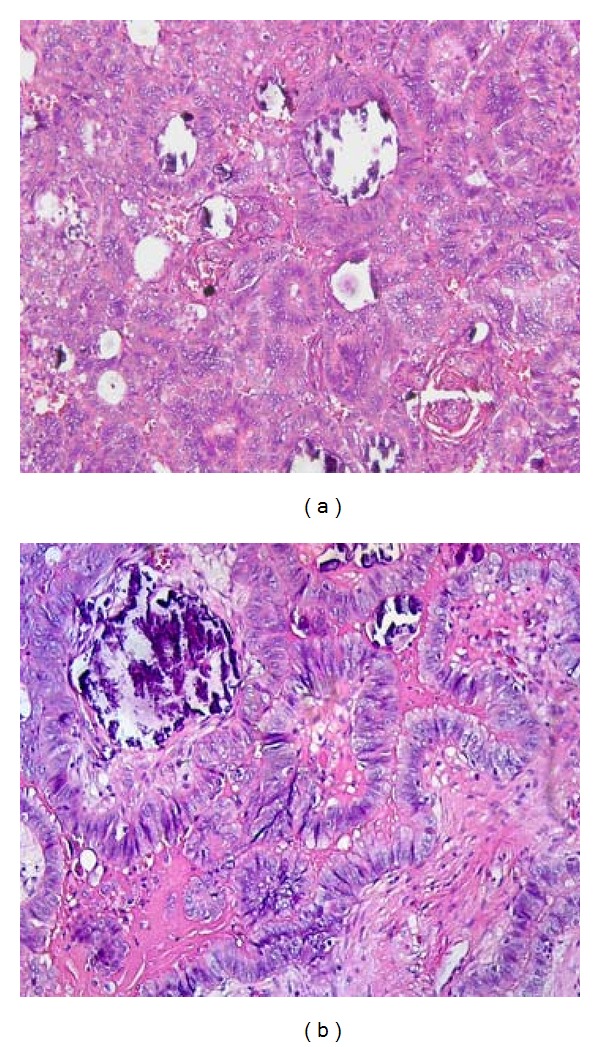
Histological H&E stain of papillary protrusion of the malignant cells filled in the lumen of rete testis. (a) (100x); (b) (400x).

Microscopically, there were hyperchromatic cuboid and columnar cells with papillary protrusions which fulfill the center hilus of testis (Figures 2 and 3). There were some calcifications embedded. The part of epididymis appeared normal with few microinfiltrations to the head of epididymis. The mass was identified to be papillary adenocarcinoma of rete testis according to the Nochomovitz and Orenstein's classifications [3]. Serum *α*-fetoprotein (AFP) and *β*-human chorionic gonadotropin (*β*HCG) were within normal limits one week after partial epididymectomy. Computerized tomography (CT) of the abdomen revealed no visible lymph node in the retroperitoneal space or pelvic cavity. He further underwent a left inguinal radical orchiectomy 1 month after the first operation. The final pathological report revealed no residual tumor in testis, epididymis, or the stump of the dissected spermatic cord. There was no further adjuvant treatment such as radiotherapy or chemotherapy. He had been followed by serum AFP, *β*HCG, and abdominal CT annually without evidence of tumor recurrence for at least 48 months.

## 3. Discussions

To our best knowledge, papillary adenocarcinoma is a rarely extratesticular malignancy with only about 60 cases reported in the literatures [1, 4]. It tends to be found in the aged patients with the peak incidence of 70 years (ranged from 17 to 91 years) [1, 4, 5]; only 6 patients were younger than 30 years old [4, 5]. The most reported cases were white and occasionally seen in Africans and oriental persons.

The exact etiology of this tumor remains unknown. The most common symptoms of papillary adenocarcinoma included scrotal mass, indurations, pain, and swelling, which mimicked an inflammation. The misleading symptoms may delay the diagnosis and adequate treatment. It is difficult to make differential diagnosis with other extratesticular lesions because adenocarcinoma of rete testis may present with epididymitis, cryptorchidism, hydrocele, or inguinal hernia [6–10]. There is no specific or sensitive marker including APF and *β*HCG to detect the tumor earlier. The diagnosis of adenocarcinoma of rete testis was often made by the pathologist after surgery. There are 5 criteria for the diagnosis of a primary tumor of the rete testis proposed by Nochomovitz and Orenstein: (1) absence of histologically similar extrascrotal tumor, (2) tumor centered on testicular hilus, (3) morphology incompatible with any other type of testicular or paratesticular tumor, (4) demonstration of a transition between the unaffected rete testis and the tumor, and (5) a predominantly solid appearance [3]. Our pathological pictures fulfill these criteria.

The prognosis of papillary adenocarcinoma seemed poor in the earlier reports. Cancer cells may invade locally, metastasize via lymphatics to the para-aortic and iliac lymph nodes, or hematogenously spread to the sites of lung and bone [1]. The involvement of pelvic lymph node had been reported [3]. The treatment of choice is surgery which includes radical orchiectomy and retroperitoneal lymph node dissection (RPLND). Chemotherapy and radiotherapy only have a limited benefit to patients' survival time [3]. These were 40~50% patients died within the first year after diagnosis [3, 5, 7]. Three- and five-year disease-free survival was 49% and 13%, respectively [7]. Only 37% of the reported patients had an average 8-month tumor-free survival after diagnosis [3]. Unlike other malignancies, the stage cannot affect the prognosis or the choice of further treatment. According to the study of Sanchez-Chapado et al., the size of the tumor was the only independent predictor for the survival time [7]. Therefore, prognosis seems not affected by the methods of treatment.

Usually, the patient was a sexualactive young man with symptoms of inflamed left scrotum and lower urinary tract symptoms which were considered as a common epididymitis and urinary tract infection. The scrotal tumor was relatively small and was difficult to be differentiated with a swollen epididymis. It was lucky to identify the tumor from ultrasonography in a small size and early stage. After radical orchiectomy, the patient had at least 48-month tumor-free period. Good prognosis was probably due to the fact that our patient was young with a small and early-stage tumor. Although Sanchez-Chapado et al. suggested the benefit of RPLND [7], we did not perform RPLND to this patient because of a clear-cut end of the spermatic cord and his sexual active age. Sympathetic nerve fiber has a risk to be disrupted during this procedure. That may result in loss of seminal emission and cause ejaculation problem. We suggested close followup by serial examinations, and the result was good till now.

In conclusion, papillary adenocarcinoma of rete testis is a rare extratesticular malignancy which may have a diverse prognosis in different age. No definite predictor and tumor marker can be used to define its prognosis and method of treatment. A better prognosis may be reached if diagnosis and surgical were conducted treatment earlier.
